# Intestinal-pulmonary axis: a ‘Force For Good’ against respiratory viral infections

**DOI:** 10.3389/fimmu.2025.1534241

**Published:** 2025-03-18

**Authors:** Jianing Zhu, Zihang Huang, Ying Lin, Wenxu Zhu, Binbin Zeng, Dong Tang

**Affiliations:** ^1^ Clinical Medical College, Yangzhou University, Yangzhou, China; ^2^ Department of General Surgery, Institute of General Surgery Northern Jiangsu People’s Hospital Affiliated to Yangzhou University, Yangzhou, China; ^3^ Northern Jiangsu People’s Hospital, Yangzhou, China; ^4^ The Yangzhou Clinical Medical College of Xuzhou Medical University, Yangzhou, China; ^5^ The Yangzhou School of Clinical Medicine of Dalian Medical University, Yangzhou, China; ^6^ The Yangzhou School of Clinical Medicine of Nanjing Medical University, Yangzhou, China; ^7^ Northern Jiangsu People’s Hospital, Clinical Teaching Hospital of Medical School, Nanjing University, Yangzhou, China

**Keywords:** respiratory viral infections, gut-lung immune axis, intestinal microbiota, systemic transport of gut microbiota-derived metabolites, immune cell migration, immune factor cycling

## Abstract

Respiratory viral infections are a major global public health concern, and current antiviral therapies still have limitations. In recent years, research has revealed significant similarities between the immune systems of the gut and lungs, which interact through the complex physiological network known as the “gut-lung axis.” As one of the largest immune organs, the gut, along with the lungs, forms an inter-organ immune network, with strong parallels in innate immune mechanisms, such as the activation of pattern recognition receptors (PRRs). Furthermore, the gut microbiota influences antiviral immune responses in the lungs through mechanisms such as systemic transport of gut microbiota-derived metabolites, immune cell migration, and cytokine regulation. Studies have shown that gut dysbiosis can exacerbate the severity of respiratory infections and may impact the efficacy of antiviral therapies. This review discusses the synergistic role of the gut-lung axis in antiviral immunity against respiratory viruses and explores potential strategies for modulating the gut microbiota to mitigate respiratory viral infections. Future research should focus on the immune mechanisms of the gut-lung axis to drive the development of novel clinical treatment strategies.

## 1 Introduction

Respiratory viruses primarily invade through the respiratory tract, where they can proliferate extensively in the epithelial cells of the respiratory mucosa, causing localized infections or leading to damage in other tissues and organs (1). Common viral families include Orthomyxoviridae, Paramyxoviridae, and Coronaviridae, along with less common families like Togaviridae and Picornaviridae. Respiratory viral infections have long posed a significant public health challenge. In 2015 alone, respiratory syncytial virus (RSV) infections led to approximately 3.2 million hospitalizations and 59,600 deaths in children under five globally (2). Additionally, influenza viruses continue to impose a substantial global health burden, affecting approximately 8% of adults and 25% of children each year (3). While the mortality rate of seasonal influenza is generally low, severe infections caused by certain influenza strains, such as H5N1 and H7N9, can have mortality rates of 50% or higher (4). Although existing antiviral drugs and immunomodulatory therapies have demonstrated efficacy in combating respiratory viral infections, many such infections still lack specific treatments (5, 6), and approved therapies often rely on synthetic drugs that can be limited by side effects and the development of viral resistance (7, 8). There is an urgent need to identify new strategies for better controlling respiratory viral infections.

Traditional research on the immune response to respiratory viruses has primarily focused on the immune defenses of the respiratory system itself. The lungs have distinct immune defense mechanisms, including mucosal barriers, immune cells, and immune molecules (9, 10). However, recent studies have increasingly demonstrated that immune signaling pathways between the gut and respiratory system are interconnected. The gut, the largest immune organ in the body, hosts a diverse microbiota that interacts with the host’s immune system to maintain immune homeostasis (11). Research has shown that under certain pathological conditions, there is considerable overlap in the types and functions of immune cells in the gut and lungs, enabling coordinated responses to pathogen invasion (12, 13). Specific members of the gut microbiota are associated with resistance to respiratory viral infections (14), and changes in gut microbiota composition may affect susceptibility to respiratory viruses and the progression of disease (15). This complex biological and immunological interaction between the gut and lungs is referred to as the “gut-lung axis.” With an in-depth understanding of the mechanism of action of the gut-lung axis in the fight against respiratory viral infections, it is expected to regulate the body’s immune function, enhance the resistance to respiratory viral infections, and attenuate the inflammatory response and pathological damage by intervening in the interaction of the gut-lung axis, thus providing a new way of thinking and a potential target for the prevention and treatment of respiratory viral infections.

## 2 Definition of the gut-lung axis

The lungs and gut both belong to the common mucosal immune system (CMIS), serving as critical defense organs that protect the body from pathogen invasion through both innate and adaptive immune mechanisms. Research indicates that stimulation of one organ can affect the immune responses of another, forming what is referred to as the gut-lung cross-talk pathway (16, 17), also known as the gut-lung axis. Gut-Lung Axis refers to the complex network of interactions between the gut and lungs through the nervous, endocrine, and immune systems. This concept emphasizes the close connection between two organs, particularly in terms of their roles in inflammatory response and immune regulation. This connection spans anatomical, microbiological, and immunological dimensions.

From an anatomical perspective, although the lungs and gut are distant from each other, there are potential anatomical links that reinforce the existence of the gut-lung axis. Current studies suggest that communication along this axis may occur through the bloodstream (18, 19), lymphatic system (20), and neuroendocrine pathways (21). Microbiologically, both the lungs and gut harbor distinct microbial communities. The intestinal microbiota consists mainly of *Bacillota, Bacteroidota, Actinobacteria*, etc., and is diverse and abundant. The lung microbiota, on the other hand, is relatively simple, consisting mainly of *Pseudomonadota, Actinobacteria*, etc., and its abundance is much lower than that of the intestinal tract (22). The gut-lung axis encompasses interactions between host and microbiota, as well as between different microbial communities, playing a key role in maintaining host homeostasis and contributing to disease progression. For instance, in patients with bronchopulmonary dysplasia (BPD), the relative abundance of *Bacillota* is significantly lower than the non-BPD group. At the genus level, *Clostridium sensu stricto 1* was sig nificantly lower in the BPD group. However, *Veillonella*, *Roseburia*, *Micrococcus*, *Xanthomarina* were significantly enriched in the BPD group. Gut dysbiosis may contribute to BPD progression by altering immune function and metabolism (23). From an immunological standpoint, the lungs and intestines share many common immune cells, such as tissue-resident memory T cells (TRMs) (24), Invariant natural killer T (iNKT) (25), Mucosal-associated invariant T (MAIT) (26–28). In addition, the microbiota in both the lungs and gut can influence the development, maturation, and function of immune cells, thereby regulating both local and systemic immune responses. Studies have shown that gut microbiota can modulate immune cell composition and function through the production of short-chain fatty acids (SCFAs) and other metabolites (29). Supplementing specific gut microbiota may help reestablish and restore the host’s immune response (30).

## 3 Physiological basis of the gut-lung axis

The high degree of conservation of respiratory virus-recognizing receptors in intestinal and lung tissues, as well as the remarkable similarity in the mechanisms of antiviral immune response in these two organs, together form the molecular and immunological basis of the gut-lung axis.

### 3.1 Similarities between the gut and lungs in respiratory virus recognition receptors

The innate immune system serves as the first line of defense against pathogen invasion, with receptors that can specifically recognize pathogen-associated molecular patterns (PAMPs) (31, 32). In mammals, the innate immune response is triggered by host pattern recognition receptors (PRRs) that detect PAMPs. When PRRs such as Toll-like receptors (TLRs) and RIG-I-like receptors (RLRs) recognize PAMPs (33–35), they initiate signaling pathways that activate the host cell’s defense mechanisms (36). This recognition leads to cellular responses such as regulating transcription factors essential for the production of interferons(IFNs) and cytokines, increasing expression of MHC class II and inducing expression of the costimulatory molecules CD40, CD80 and CD86, aimed at neutralizing pathogens and activating other defense mechanisms (37). Both the gut and lung immune systems harbor similar virus recognition receptors, forming a critical component of the host’s innate immune defense. **Table 1** summarizes in detail the pulmonary and intestinal shared viral recognition receptors in common respiratory viral infections.

**Table 1:** Generalization of viral recognition receptors shared by lung and intestine and similar immune mechanisms in common respiratory viral infections.

Respiratory Viruses	Shared Receptor Types	Similar Signaling Pathways Activated	References
SARS-CoV-1 SARS-CoV-2	ACE-2	Angiotensin II → Ang (1-7)	(80–82)
Flu Virus	RLRs	IFN ↑	(57–59)
RSV	TLRs	MyD88↑ TRIF↑ IFN ↑	(76, 83)
Adenovirus	Coxsackievirus-Adenovirus Receptor (CAR)	Leucocyte recruitment tissue remodeling	(84–86)
Human Metapneumovirus (HMPV)	Acetylheparin Sulfate Proteoglycan (HSPG)	Integrin avβ 1↑	(87)
Human Parainfluenza Virus (HPIV)	Sialic Acid	Virus attachment and entry into host cells	(88)
Human Microvirus B19 (B19V)	Globotetraosylceramide (Gb4Cer)	nonstructural (NS)1 protein↑	(89, 90)

A→B: metabolizes A into B.

A↑: increased expression levels of A.

#### 3.1.1 The role of SARS-CoV-2 and ACE-2 receptors in the lungs and gut

The COVID-19 pandemic, caused by SARS-CoV-2, has demonstrated high transmissibility and infectivity. The angiotensin-converting enzyme 2 (ACE-2) receptor plays a crucial role in this process, serving as the entry point for SARS-CoV-2 into cells, while also contributing to the maintenance of lung and gut health (38).

SARS-CoV-2 invades host cells by binding to the ACE-2 receptor. Studies have shown that ACE-2 receptors are widely distributed in alveolar cells, explaining the virus’s ability to cause severe respiratory disease (39). Additionally, biopsies of the stomach, duodenum, and rectum from infected patients have revealed the presence of both SARS-CoV-2 and ACE-2 receptors in gastrointestinal tissues (40–43), indicating that the gut is also a potential site of viral entry (44–46). Following infection, gastrointestinal symptoms such as nausea, vomiting, and diarrhea may occur in addition to the common respiratory symptoms of cough, shortness of breath, and loss of smell. Research has found that up to half of COVID-19 patients report gastrointestinal symptoms (47). SARS-CoV-2 invades host cells by binding its spike glycoprotein (S protein) to ACE-2 receptors on the surface of host cells (48–50). This invasion occurs not only in the lungs but also in the gut. *In vitro* experiments have confirmed that SARS-CoV-2 can efficiently enter and replicate in colonic epithelial cells (51, 52),This study demonstrates that ACE-2 receptors consistent with the lung can also be expressed in the gut, providing binding sites for viral infections.

In summary, the high expression of ACE-2 receptors in both the respiratory and gastrointestinal systems provides a biological explanation for the elevated infection rates of SARS-CoV-2 in these two systems, revealing a similarity in the virus recognition mechanisms of the gut and lungs.

#### 3.1.2 The role of influenza virus and RLRs in the lungs and gut

Influenza viruses, belonging to the Orthomyxoviridae family, are classified based on specific combinations of their surface proteins, hemagglutinin (HA) and neuraminidase (NA), with 18 HA and 11 NA subtypes identified so far (53). During infection, the HA on the viral surface binds to sialic acid receptors on host cells, allowing the virus to attach to target cells. Subsequently, through a process mediated by clathrin-mediated endocytosis (CME), the virus enters host cells via specific N-linked glycoproteins (54, 55).

PRRs detect viral genetic material entering the cell, which in turn initiates a series of signaling cascades to effectively inhibit viral replication and remove the virus before the infection becomes severe, protecting the organism from further aggression (56). Among these PRRs, the RLR family—including RIG-I, MDA5, and LGP2—plays a crucial role. Studies show that both RIG-I and MDA5 are capable of recognizing RNA viruses in the epithelial cells of the gut and lungs, detecting viral RNA in infected cells (57–59). The activation of RIG-I is essential for the production of IFNs in response to viruses such as paramyxoviruses, influenza viruses, and Japanese encephalitis viruses (60, 61), while MDA5 is particularly important for detecting small RNA viruses (62, 63).

RLRs, as RNA sensors localized in the cytoplasm, are widespread in human cells, particularly in epithelial cells in direct contact with the external environment, such as the gut and lungs. Activation of RLR can stimulate an antiviral response by initiating cellular autophagy, effectively inhibiting the process of viral replication before it occurs (64). This early defense mechanism is critical for halting viral spread and mitigating disease symptoms.

#### 3.1.3 The role of RSV and TLRs in the lungs and gut

RSV is a common virus, posing a significant health threat, particularly to infants (65–67). TLRs, key components of the innate immune system, recognize PAMPs and trigger immune responses (68, 69). Recent studies have increasingly focused on the role of RSV and TLRs in regulating immune responses in both the lungs and gut. As vital organs in the body, the immune balance of the lungs and gut is crucial for overall health. RSV infection not only affects the lungs but may also impact gut immunity, with TLRs serving as critical mediators in this process.

Once RSV infects the lungs, it can influence gut immune function via the bloodstream or neuroendocrine pathways. RSV induces inflammation and recruits immune cells. Viral infection damages the respiratory epithelium, releasing cytokines and chemokines that attract immune cells such as neutrophils, macrophages, and lymphocytes to the site of infection (70, 71). Furthermore, lung inflammation caused by RSV can lead to systemic inflammatory response syndrome (72). This inflammation may compromise the integrity of the gut barrier by altering the expression of junction proteins in intestinal epithelial cells (IECs) (73), allowing pathogens and toxins easier entry into the body, which exacerbates inflammation and immune dysregulation (74, 75), ultimately affecting lung immune function.

The connection between TLRs in lung and gut immune regulation involves several key aspects: First, TLRs in both organs including TLR3, TLR4, TLR7/8 and TLR2/6 can recognize different components of RSV and initiate immune responses (76). Second, TLRs regulate the production of cytokines and chemokines (77, 78), affecting the recruitment and activation of immune cells, thereby facilitating immune crosstalk between the lungs and gut. Finally, TLRs modulate gut barrier function, controlling the entry of pathogens and toxins into the bloodstream, which in turn influences lung immune function (79).

### 3.2 Similarities in antiviral immune mechanisms between the gut and lungs

**Figure 1:**
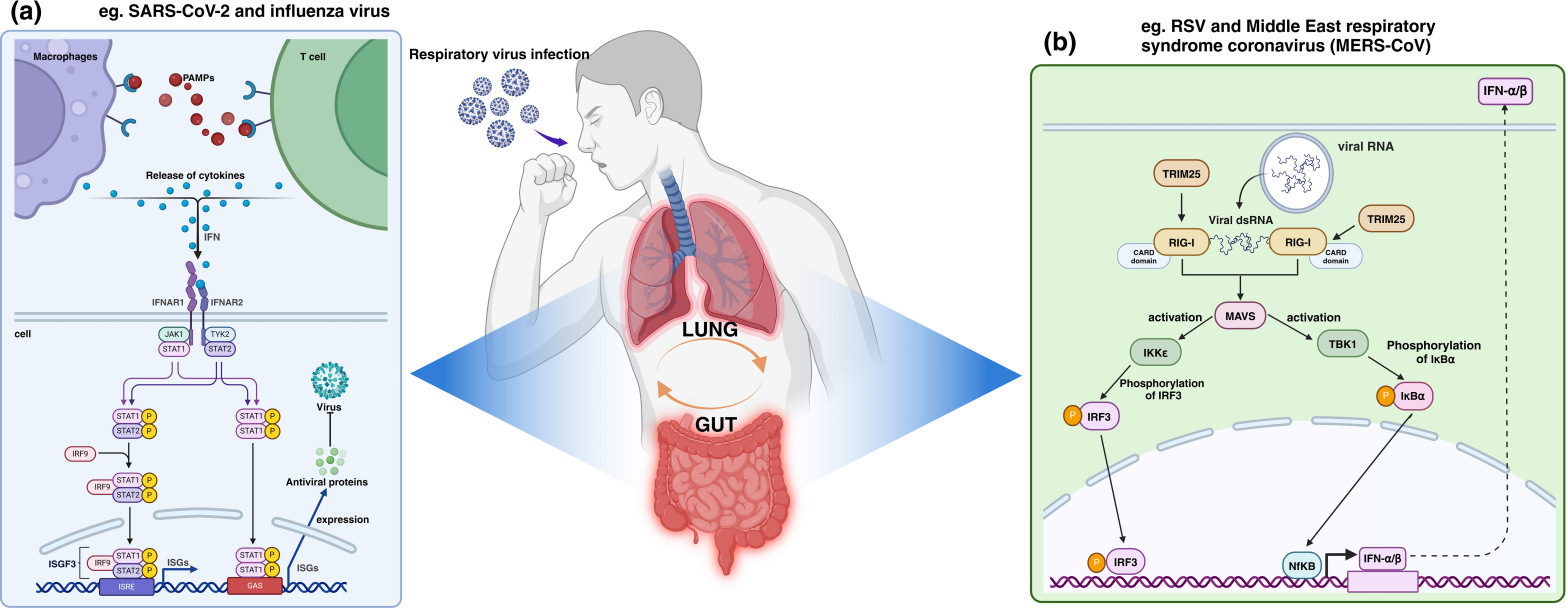
Human antiviral immune mechanism shared by the lungs and intestines, taking ISGs and TRIM25 antiviral immune mechanism as examples. As shown in section **(a)**, during the infection process of SARS-CoV-2 and influenza virus, etc., ISGs such as Janus kinase signal transduction and transcription activators (JAK-STAT) are significantly upregulated, initiating a broad range of antiviral effects by ISGs. In section **(b)**, RNA from RSV and MERS-CoV, etc., triggers a conformational change in RIG-I, which TRIM25 binds to and oligomerizes, activating the MAVS to initiate the expression of a series of antiviral genes. PAMPs, Pathogen-associated molecular patterns; IFN, Interleukin; IFNAR, Interleukin receptor.

In addition to their similarities in viral recognition mechanisms, the gut and lungs exhibit highly consistent immune strategies in response to viral invasion. Both rely on the expression of interferon-stimulated genes (ISGs) and the ubiquitination pathway mediated by TRIM25 to mount antiviral immune responses, effectively defending against viral infections. This coordinated immune action further underscores the critical role of the “gut-lung axis” in antiviral immunity. **Table 1** summarizes in detail the similar immune mechanisms exhibited by the lungs and intestines in common respiratory viral infections. **Figure 1** lists two similar immune pathways exhibited by the gut and lungs under common respiratory virus infections.

#### 3.2.1 ISGs and antiviral immunity

IFNs, as the first line of defense in antiviral immunity, play a key role in inhibiting viral replication, promoting apoptosis, and enhancing the activity of immune cells by inducing the expression of ISGs. During infections in both the gut and lungs, ISGs such as Janus kinase signal transducer and activator of transcription (JAK-STAT) are significantly upregulated, demonstrating the widespread applicability of these genes in antiviral immunity across these two organs. Such mechanisms have been implicated in both the SARS-CoV-2 and influenza virus pathways of human infection (91, 92).

Upon recognition of PAMPs, host cells rapidly produce and secrete IFNs (93). IFNs, which regulate the host’s defense against pathogens, are classified into Type I, II, and III, with Type I IFNs (including IFN-α and IFN-β) being particularly critical in antiviral responses (94). Type I IFNs bind to cell surface interferon receptors (IFNAR), activating intracellular Janus kinases (JAKs) and signal transducers and activators of transcription (STATs).

The activation of the JAK-STAT signaling pathway is a key step in IFN signal transduction. When IFNs bind to IFNAR, JAKs are recruited and phosphorylated, subsequently activating STATs. Phosphorylated STATs form dimers that translocate to the nucleus, binding to specific DNA sequences to initiate ISG transcription (95). The expression of ISGs triggers broad antiviral effects by inhibiting viral entry into cells and blocking viral replication and assembly (96).

ISGs encode antiviral proteins such as Mx protein, protein kinase R (PKR), and 2’,5’-oligoadenylate synthetase (OAS) (97–99), which effectively suppress viral replication and transmission. Additionally, ISGs participate in regulating the activation, proliferation, and differentiation of immune cells, and they promote inflammatory responses, thereby limiting pathogen infection and spread on multiple fronts (100, 101). Therefore, ISG expression is a core component of the host’s defense mechanism against pathogens, playing a vital role in maintaining the balance between host and pathogen.

#### 3.2.2 TRIM25-mediated ubiquitination pathway and RIG-I antiviral signaling

TRIM25, as an E3 ubiquitin ligase, is able to activate RIG-I via K63-strand ubiquitination, triggering the synthesis and secretion of type I interferon and thus initiating an antiviral immune response (102, 103). Activation of this pathway significantly enhances host antiviral immunity, which is one of the important mechanisms for resisting the invasion of respiratory viruses such as RSV and Middle East respiratory syndrome coronavirus (MERS-CoV) (102, 104).

In antiviral innate immunity, RIG-I acts as a key RNA deconjugating enzyme and plays an important role in recognizing viral RNA (105). When viruses invade host cells and release their RNAs, RIG-I specifically recognizes these “non-self” RNAs, triggering a series of complex signaling cascades. Recognition of viral RNAs by RIG-I leads to a conformational change, exposing the hidden CARD domains(CARDs) (106, 107), which provides a binding site for TRIM25 recruitment. After TRIM25 recognizes and binds to the CARDs of RIG-I (108), it acts as an E3 ubiquitin ligase, which catalyzes the attachment of K63-linked ubiquitin chains to specific lysine residues on RIG-I. This form of ubiquitination does not target the protein for degradation but instead modulates signal transduction (109), altering the biochemical properties of RIG-I and promoting its oligomerization. This oligomerization is crucial for the full activation of RIG-I, enabling it to interact with the downstream mitochondrial antiviral signaling protein (MAVS) (110).

MAVS serves as the central node in antiviral signal transduction, activating several kinases, including TBK1 and IKKϵ (111). These kinases, in turn, phosphorylate transcription factors such as IRF3 (interferon regulatory factor 3) and NF-κB, driving them into their active states (112). Once activated, these transcription factors translocate into the nucleus and initiate the expression of a suite of antiviral genes, including type I interferons (IFN-α and IFN-β) and pro-inflammatory cytokines (113).

IFNs propagate between infected and neighboring cells in an autocrine and paracrine manner, amplifying antiviral signals through the JAK-STAT signaling pathway, inhibiting viral replication, and inducing the expression of a range of antiviral proteins in host cells. Thus, the TRIM25-mediated ubiquitination pathway and the RIG-I antiviral pathway are a complex and sophisticated regulatory network that ensures that the host is able to rapidly and efficiently initiate an immune response against viral infection.

## 4 Immunological linkage mechanisms between the gut and the lungs

In recent years, a growing body of research has highlighted the close relationship between the gut and the lungs in terms of immune mechanisms and functions. Pulmonary diseases are often accompanied by intestinal damage, and conversely, intestinal diseases can also trigger pathological changes in the lungs. For example, influenza virus infection can cause gastrointestinal symptoms such as vomiting and diarrhea (114), HMPV can modulate intestinal adaptive immunity despite the absence of viral expression in the gut (115). Moreover, patients with inflammatory bowel disease (IBD) frequently develop respiratory diseases such as asthma and chronic obstructive pulmonary disease (COPD) (116). This bidirectional relationship underscores the significance of the “gut-lung axis.” This section further explores these mechanisms in the context of the gut-lung axis and their impact on respiratory viral infections.

### 4.1 Migration of intestinal microbiota

It is well known that the human body harbors a vast array of microorganisms, including bacteria, fungi, viruses, and archaea. Among these, the gut microbiota is the most densely populated, primarily consisting of species such as *Bifidobacterium*, *Lactobacillus*, and *Escherichia coli (* 117). As a crucial component of the intestinal barrier, the gut microbiota plays an essential role in digesting food, synthesizing vitamins, regulating the immune system, and defending against pathogens (118).

Although the microbiota of the gut and lungs are distributed in different anatomical locations, they are closely linked through the “gut-lung axis.” The translocation of microbiota is one of the key mechanisms underlying this connection. In 2023, a study by Jayanth Kumar Narayana et al. found that in stable bronchiectasis, the microbiota community exhibited significant gut-lung interactions. The translocation of bacteria between the gut and lungs may be associated with increased overall severity of bronchiectasis, suggesting that microbial migration between these organs is related to the disease (119). Studies have shown that some microbiota can migrate to other organs and tissues, such as the brain (120), muscles (121), and lungs. This migration can influence local tissues and may even trigger systemic inflammatory responses, further exacerbating disease progression. Acute respiratory distress syndrome (ARDS) is an acute and diffuse pulmonary inflammation and a common cause of respiratory failure, often secondary to various respiratory viral infections, such as influenza A virus (IAV). In 2016, Robert P. Dickson and colleagues conducted sequencing of bacterial communities in bronchoalveolar lavage (BAL) specimens from ARDS patients and found that certain Bacteroides operational taxonomic units (OTUs) absent in healthy lungs were highly consistent with those found in four anaerobic genera from the gut, and these were associated with the severity of acute systemic inflammation (122). Similarly, in 2023, Dusanka Popovic et al. used the *fungus A. fumigatus* to induce pulmonary inflammation and discovered that the disruption of pulmonary homeostasis facilitated the migration of new bacterial species to the lungs, with 41.8% of the bacteria also present in fecal samples, indicating a degree of gut microbiota translocation to the lungs (123). These findings support the existence of the “gut-lung axis” and suggest a bidirectional relationship between lung inflammation and gut microbiota dysbiosis.

### 4.2 Regulatory role of intestinal microbiota

**Figure 2:**
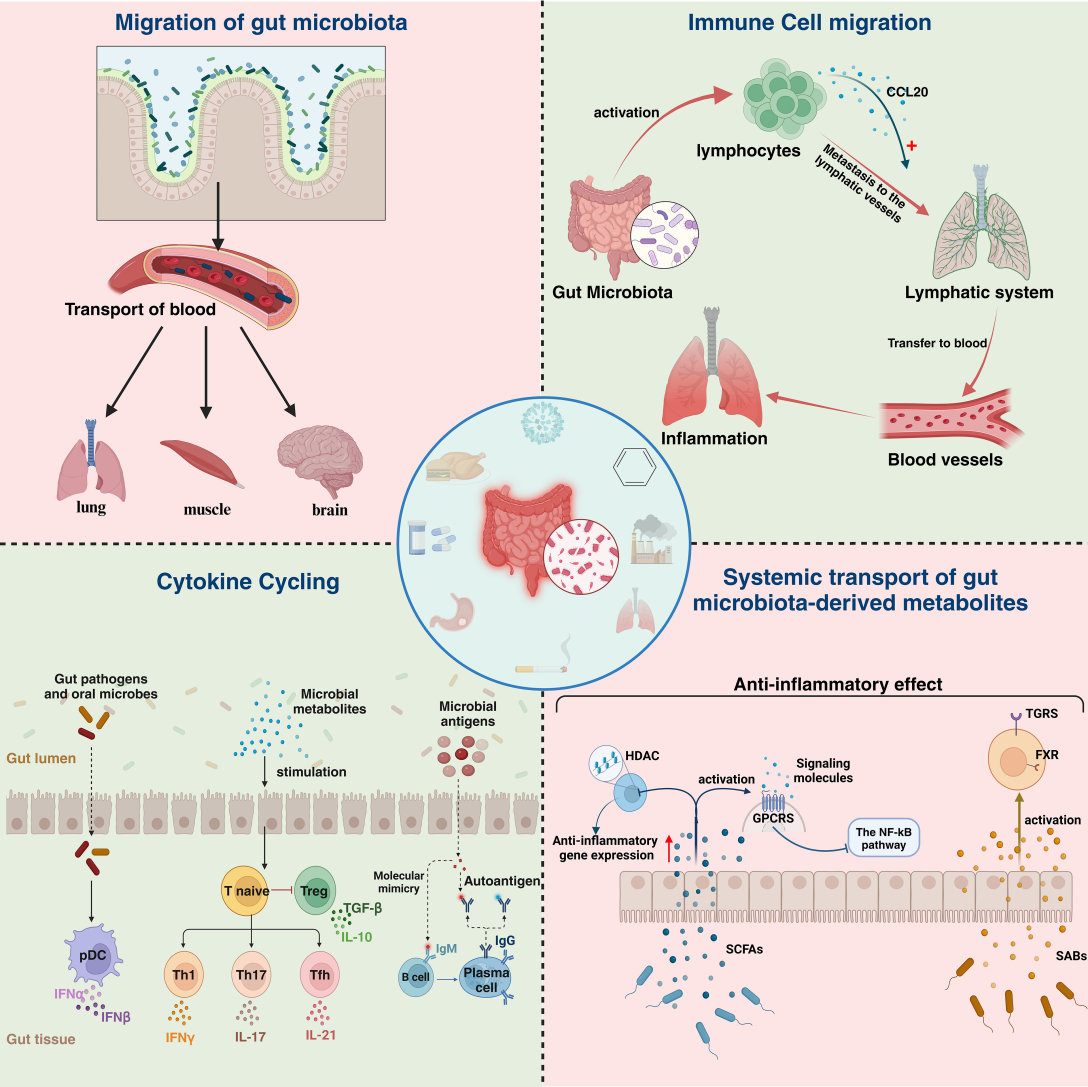
Specific manifestations of lung and intestinal immune correlations. Lung and intestinal immune relevance is manifested in four main areas: Migration of gut bacteria, immune cell migration,cytokine recycling and systemic transport of gut microbiota-derived metabolites. HDAC, histone deacetylases; GPCRS, G protein-coupled receptors;SCFAS, short-chain fatty acids; TGRS, traditional gender roles; FXR, Farnesoid X receptor ;SABs, Secondary bile acids.

More and more studies have proved that microbiota can not only migrate directly to target organ to play an immune role, but also regulate the immune function of the body through a variety of ways, such as systemic transport of gut microbiota-derived metabolites, immune cell migration, and cytokine cycling (**Figure 2**). 2022, Xiaowu Baiet al. found that cigarette smoke can directly lead to the disruption of the intestinal microbiota of the mouse, which can lead to the impairment of the intestinal barrier and enhance the expression of oncogenic signals and pro-inflammatory genes (124). This suggests that the disruption of intestinal microbiota may be the starting point for the regulation of immune function by intestinal microbiota. Similarly, IAV, COVID-19 and other respiratory viruses also showed disturbances in the intestinal microbiota (114, 125), which provides a target for us to further investigate the role of intestinal microbiota regulation in the context of respiratory virus infection.

#### 4.2.1 Systemic transport of gut microbiota-derived metabolites

Metabolites produced by the gut microbiota, such as SCFAs and secondary bile acids, can influence distant organs, including the pulmonary immune environment, via the bloodstream, thereby enhancing the efficiency of antiviral immune responses. This systemic transport of gut microbiota-derived metabolites not only alters the host’s energy metabolism but also regulates immune responses, bolstering the host’s resistance to respiratory viral infections. The alterations may be associated with changes in the host’s defense mechanisms driven by microbial metabolites. For instance, a study by Michael C. Abt et al. in 2012 demonstrated that the responsiveness of macrophages to type I and II IFNs was impaired in ABX-treated mice, which consequently reduced their ability to control viral replication. This finding indicates that commensal bacteria influence the activation threshold of widely utilized innate antiviral pathways, such as the IFN signaling pathway (126). Similarly, a study published in 2023 by Junling Niu et al. revealed that the acetate produced by *Bifidobacterium pseudolongum* NjM1 promotes the interaction between NLRP3 and the MAVS by binding to the GPR43 receptor on the host cell surface. This interaction subsequently activates the TBK1/IRF3 pathway, driving the production of type I IFNs (127). **Table 2** summarizes in detail the immune impact exerted by several common microbial metabolites in the fight against respiratory viral infections.

**Table 2:** Immune effects of different microbial metabolites in the fight against respiratory viral infections.

Metabolite	Primary Sources	Impact	References
Butyrate	Dietary fiber fermentation by anaerobic bacteria	Enhances barrier via GPR109A/AMPK; induces Treg via HDAC inhibition.	(129)
Propionate	Dietary fiber fermentation by anaerobic bacteria	Suppresses DC inflammation; promotes M2 macrophage polarization.	(132, 133)
Acetate	Dietary fiber fermentation by anaerobic bacteria	Boosts IgA via B-cell glycolysis; inhibits neutrophil chemotaxis.	(134, 135)
Bile Acids	Metabolism of bile acids by gut microbes	Modulation of intestinal inflammation and reduction of macrophage pro-inflammatory responses via FXR and TGR5 receptors	(138, 143)
Indoles	Gut bacteria metabolize tryptophan	Reduction of pro-inflammatory cytokines through aromatic hydrocarbon receptor (AhR) regulation of intestinal barrier integrity and immunomodulation	(144–146)
Lactic Acid	Lactobacillus fermentation produces	Regulates the intestinal internal environment, reduces the inflammatory response and promotes the expression of anti-inflammatory cytokines	(147)
LPS	Gram-negative bacteria	Activation of TLR4 receptor triggers a strong pro-inflammatory response leading to immunopathology	(148)
TMAO	Enterobacteriaceae	lead to inflammation	(149)
Phenylacetyl Glutamine (PAGln)	Christensenellaceae and other microbiota	Significantly reduces inflammatory mediators	(150)

SCFAs, the primary metabolites generated by the fermentation of dietary fiber by gut microbiota, play a pivotal role in maintaining epithelial barriers and programming immune cells under homeostatic conditions. Butyrate, in particular, inhibits histone deacetylases (HDACs) through various pathways, thereby enhancing the expression of anti-inflammatory genes (128). Butyrate indirectly promotes the differentiation of effector T cells (Teff) by increasing the acetylation levels of histone H3K9 in chromatin. Additionally, by blocking the deacetylation of the promoter regions of exhaustion-related genes (such as PD-1 and TIM-3), butyrate inhibits the exhausted phenotype of CD8^+^ T cells in chronic immune responses. At the metabolic level, butyrate can reshape the energy metabolism of CD8^+^ T cells, supporting cell survival in a low-glucose microenvironment and restricting the excessive activation of Teff and inflammatory damage (129). Wei Wang and colleagues found that butyrate induces transcriptional changes via HDAC inhibition, ultimately reducing the expression of cFLIP and IL-10, thereby activating the NLRP3 inflammasome to trigger pro-inflammatory processes (130). Additionally, butyrate regulates the number and function of effector cells such as regulatory T cell (Treg), T helper cells 1 (Th1), and T helper cells 17 (Th17) (131), helping to maintain immune balance, reduce inflammation, and preserve gut homeostasis. Propionate primarily modulates immune responses via the GPR43 receptor, suppressing IL-12 and TNF-α secretion in dendritic cells while promoting the expansion of gut-homing Tregs, thereby reinforcing a localized anti-inflammatory microenvironment (132). Additionally, propionate induces macrophage polarization toward an M2 anti-inflammatory phenotype through HDAC inhibition (133).Acetate, acting as a ligand for GPR43/GPR41, enhances IgA secretion by activating systemic transport of gut microbiota-derived metabolites (e.g., upregulated glycolysis) in B cells, thereby strengthening mucosallayer pathogen clearance (134). It also mitigates inflammatory damage to the epithelial barrier by suppressing neutrophil chemotactic factors (CXCL1/CXCL2) (135). SCFAs bidirectionally regulate the efficiency of memory formation and the persistence of immune responses in antigen-activated CD8^+^ T cells through G protein-coupled receptors (GPCRs) and monocarboxylate transporters (MCTs/SMCTs). In the GPCR pathway, SCFAs bind to GPR41/GPR43 to activate AMPK and inhibit mTORC1, thereby promoting the shift of CD8^+^ T cells toward a memory precursor phenotype driven by oxidative phosphorylation (OXPHOS) and fatty acid oxidation (FAO), which is accompanied by enhanced mitochondrial biogenesis and fatty acid uptake. Meanwhile, the influx of SCFAs mediated by MCTs/SMCTs reinforces metabolic adaptation by inhibiting HDAC activity and synergistically amplifying OXPHOS metabolic advantages through GPCR signaling (136). For instance, SCFAs in renal cells reduce tumor necrosis factor-α (TNF-α)-induced MCP-1 expression through a GPR41/43-dependent pathway, inhibiting the phosphorylation of p38 and JNK, thereby indirectly suppressing the NF-κB pathway and mitigating inflammation (137). Although the precise mechanisms of this inhibition vary across different cells and tissues, they all involve regulating immune responses, reducing inflammation, and improving disease states. Therefore, SCFAs possess potent anti-inflammatory properties.

Secondary bile acids, which are metabolites of bile acids by the gut microbiota, have been shown to play an essential role in regulating immune responses in both the lungs and the gut. Farnesoid X receptor (FXR) and G-protein-coupled bile acid receptor 5 (TGR5) are two important bile acid receptors that are key to regulating gut inflammation and suppressing the pro-inflammatory responses of macrophages. FXR primarily influences the gut environment by regulating bile acid metabolism, with its high expression in the gut and liver making it crucial for maintaining intestinal homeostasis. Activation of FXR can suppress inflammation, protect the intestinal barrier, fight bacterial infections, and reduce oxidative stress (138). For example, FXR activation stabilizes the binding of the corepressor NCoR at the IL-1β promoter, thereby inhibiting NF-κB-dependent gene expression. Additionally, it increases the expression of I-BABP in the intestine while reducing mRNA levels of interleukin-1β(IL-1β), interleukin-2 (IL-2), interleukin-6 (IL-6), TNF-α, and IFN-γ, thereby alleviating disease severity (139). TGR5, a cell surface receptor, responds to stimulation by secondary bile acids and plays a role by directly suppressing the pro-inflammatory responses of macrophages. Specifically, TGR5 deficiency exacerbates inflammation, whereas TGR5 activation inhibits NLRP3 inflammatory vesicle activation and M1-type macrophage polarization (140). It has been shown that activation of the bile acid-TGR5 axis prevents influenza virus infection or inhibits the inflammatory response following influenza virus infection (141).

Similar to the gut, microbial metabolites in the lung also exert significant impacts on the host immune system. A study by Jingli Li et al. in 2020 demonstrated that exposure to PM2.5 significantly altered the richness, evenness, and composition of the lung microbiota, and disrupted the levels of pulmonary metabolites such as valine, acetate, and fumarate. These changes not only affect normal energy metabolism in the host but also predispose to inflammation in distant organs (142).

#### 4.2.2 Immune cell migration

The gut contains 70-80% of the body’s immune cells, which play critical roles in maintaining gut homeostasis, defending against pathogens, and preserving mucosal barrier function. For example, group 3 innate lymphoid cells (ILC3s) secrete cytokines such as IL-22 to enhance intestinal barrier integrity and exert anti-inflammatory effects (151). Macrophages, on the other hand, contribute to post-inflammatory recovery through key signaling pathways such as NF-κB, JAK/STAT, and PI3K/AKT, as well as specific microRNAs like miR-155 and miR-29 (152).

In the gut, there is a complex regulatory and supportive relationship between the immune system and the gut microbiota. Under the stimulation of gut microbes, immune cells can become activated and exert immunosurveillance functions throughout the body. A study by Seohyun Byun et al. in 2024 demonstrated that colonic Tregs, induced by microbiota, exhibit strong suppressive abilities and higher IL-10 levels, further supporting the wide-ranging regulatory role of gut microbiota in immune function (153). Recent studies have revealed that gut microbiota metabolites can migrate to the lungs, interact with GPCRs on the surface of alveolar macrophages (AMs), and activate the AMPK-mTOR signaling axis, thereby enhancing the metabolic adaptability and antiviral response capacity of AMs. Additionally, microbial signals can strengthen the rapid recognition and phagocytic clearance of influenza virus by AMs through the TLR-MyD88 pathway. These findings indicate that the gut microbiota can enable AMs to control viral replication and suppress excessive inflammation in the early stages of infection (154). Moreover, SCFAs can inhibit the differentiation of Ly6C^+^ monocytes into an inflammatory phenotype and promote their differentiation into patrolling monocytes with tissue repair functions, thereby maintaining the local replenishment pool of AMs (155). As early as 1979, John Bienenstock hypothesized based on experimental results that the mucosal immune system might function as a system-wide “organ,” with immune cells in various mucosal tissues interacting with one another. Since the gut and lungs both belong to the CMIS, gut microbiota can influence pulmonary immunity through the “gut-lung axis” by promoting immune cell migration (156). Immune cell migration mainly occurs via two pathways: the lymphatic system and the bloodstream. First, immune cells activated by gut microbiota travel through the lymphatic system into the thoracic duct and then enter the bloodstream, spreading throughout the body, including the lungs (157). Second, gut microbiota can stimulate intestinal immune cells to release specific chemokines (such as CCL20) (158), which guide immune cells to migrate to the lungs, aiding in strengthening pulmonary immune defense. In the lungs, these immune cells enhance local immune responses, helping to fend off respiratory viral infections.

Similar to the gut, immune cells in the lung also exhibit migration to the intestine. For instance, a study by Ruane D et al. found that pulmonary CD103^+^and CD24^+^CD11b^+^ dendritic cells (DCs) induce IgA class-switch recombination (CSR) in B cells via T cell-dependent or -independent pathways. This process promotes the migration of B cells to the intestine, where they exert protective effects (159).

#### 4.2.3 Cytokine circulation

In response to infection, immune cells release cytokines, which are crucial signaling molecules. For example, upon activation by enteric pathogens such as *Staphylococcus aureus* and *Toxoplasma gondii*, plasmacytoid dendritic cells (pDCs) can produce a variety of cytokines, including IFNα and IFNβ (160). The PAMPs expressed by pathogens are recognized by PRRs expressed by IECs, which trigger immune responses by inducing various pro-inflammatory cytokines, chemokines, and type I interferons. This process further activates B and T lymphocytes to initiate humoral immunity (161). Meanwhile, the gut microbiota can influence the host’s cytokine profile through various mechanisms, thereby regulating immune responses in distant tissues (162). In particular, cytokine circulation in the gut-lung axis is considered a key factor in maintaining immune coordination between the two organs.

Gut microbiota can regulate immune function by influencing cytokine production through several pathways. For example, microbial metabolites, such as secondary bile acids, can guide the differentiation of monocytes, reducing the secretion of IL-12 and TNF-α (163). Additionally, microbe-associated molecular patterns (MAMPs), which are conserved molecular structures in bacteria, fungi, and viruses, can interact with PRRs, maintaining gut homeostasis. For instance, TLR4 recognizes lipopolysaccharide (LPS) from Gram-negative bacteria, triggering an inflammatory response and inducing the production of cytokines such as TNF-α, IL-1β, and IL-6 (164).

The effect of cytokines is not confined to the local area; they can spread throughout the body via the bloodstream. For example, in pulmonary diseases such as COPD and pulmonary fibrosis, circulating cytokines induce systemic inflammatory responses and immune regulation abnormalities (165, 166). Conversely, cytokine circulation can help the body defend against invading pathogens. A study by Chen Jiayi et al. in 2019 proposed that during influenza virus infection, gut microbiota influence IL-22 production, which enhances the integrity of the pulmonary mucosal barrier and reduces viral invasion (167). In cases of lung infection, gut microbiota can modulate the host’s cytokine profile, thereby enhancing antiviral defenses in the lungs.

Disruption of the gut microbiota can lead to increased levels of pro-inflammatory cytokines such as IL-6 and TNF-α, while the production of anti-inflammatory cytokines like IL-10 may decrease. This imbalance, transmitted through the bloodstream, can affect the lungs.During influenza virus infection, dysbiosis of the intestinal microbiota leads to elevated levels of pro-inflammatory cytokines, which exacerbate the inflammatory response in the lungs and affect the host’s immune response to influenza virus (168). Therefore, maintaining a healthy balance of gut microbiota is crucial for preventing and managing respiratory viral infections. Similarly, a study by Liu et al. demonstrated that pulmonary-derived IL-22 can promote the expression of antimicrobial peptides (such as RegIIIγ), thereby altering the composition of the gut microbiota (169). Following RSV infection, an imbalance of Th17/Treg cells occurs in the lungs of mice, leading to excessive release of IL-22 in pulmonary tissues. This IL-22 enters the systemic circulation, stimulating the expression of RegIIIγ in the gut. This process impairs the development of Th17/Treg cells in the gut, ultimately resulting in intestinal immune damage and disruption of the gut microbiota. These findings highlight that the gut-lung axis is a bidirectional pathway (169).

## 5 Regulatory role of the gut-lung axis in different respiratory viral infections

In a healthy state, the gut-lung axis maintains immune balance between the gut and lungs, ensuring normal function in both organs. However, in pathological conditions such as gut microbiota dysbiosis, viral infections, or heightened inflammatory responses, the balance of the gut-lung axis can be disrupted. This imbalance can lead to excessive or insufficient immune responses in the lungs, increasing the risk of respiratory viral infections or exacerbating their severity, potentially even affecting treatment outcomes (170).

Research has shown that during the early stages of respiratory viral infections, the gut microbiota and its metabolites can rapidly activate the host’s innate immune system, providing initial antiviral defense, regulating pulmonary inflammation, and influencing the early course of infection (171). Additionally, if the gut barrier is compromised early in infection, the translocation of endotoxins and microbes may exacerbate pulmonary inflammation, worsening the prognosis (172). In the later stages of infection, the gut microbiota plays a continuous role in regulating the host’s chronic inflammatory response. Dysbiosis in the gut-lung axis can lead to long-term imbalances, resulting in chronic lung diseases or aggravating pre-existing conditions. Late-stage infections are often accompanied by multi-organ damage and systemic inflammation, where microbial imbalance in the gut can further intensify this systemic inflammatory response. This exacerbation, through the transmission of inflammatory mediators and metabolites, can impact other organs, including the lungs (173, 174). The immune regulatory functions of the gut-lung axis vary across different respiratory viral infections.

### 5.1 Regulatory role of the gut-lung axis in influenza virus infection

The World Health Organization estimates that approximately 1 billion people worldwide are infected with the influenza virus each year, resulting in 3 to 5 million severe cases and 290,000 to 650,000 deaths. Among these, the IAV is highly transmissible, spreads rapidly, and exhibits a high mutation rate, having caused multiple global pandemics throughout history.

Research by Wang et al. demonstrated that H1N1 influenza virus (PR8 strain) infection induces gut immune damage by altering the composition of the intestinal microbiota. The CCL25-CCR9 axis mediates the recruitment of lung-derived effector CD4^+^ T cells into the small intestine and contributes to microbiota composition changes during influenza infection. These lung-derived CD4^+^ T cells, through the secretion of IFN-γ, influence both the microbiota and intestinal injury. Moreover, in conjunction with IL-15 produced by the microbiota, they promote the polarization of resident Th17 cells. Notably, a deficiency in IL-17A mitigates the immune damage induced by influenza in the small intestine (175).

During viral infection, the gut microbiota can influence pulmonary inflammation by modulating the host’s immune response. Certain probiotics, such as Lactobacillus, have been shown to enhance the host’s antiviral immunity and reduce influenza virus-induced lung inflammation. For instance, Lactobacillus rhamnosus CRL1505 has been found to mitigate lung inflammation caused by the influenza virus by modulating TLR3-mediated inflammatory responses in the lungs. This probiotic also lowers lung damage and mortality by inhibiting virus-induced inflammation-coagulation interactions (176). Additionally, Lactobacillus plantarum DK119, when administered intranasally or orally, significantly reduced body weight loss and viral load in H1N1 influenza A virus-infected mice (177), providing scientific evidence for the potential of probiotics as natural antiviral agents.

Influenza virus infection often triggers excessive activation of the host’s immune system, leading to the release of large quantities of pro-inflammatory cytokines, such as through sustained activation of the NF-κB signaling pathway. This excessive inflammatory response is a key feature of severe influenza pneumonia (178). Ichinohe et al. found that the symbiotic microbiota regulates respiratory mucosal immunity through appropriate activation of the inflammasome. Local or distal injection of TLR ligands can rescue immune deficiencies in antibiotic-treated mice. The products of the symbiotic microbiota may trigger various PRRs, stimulating the release of factors from local or systemic leukocytes, supporting the steady-state production of pro–IL-1β, pro–IL-18, and NLR proteins, thereby priming signal 1 for inflammasome-dependent cytokine activation. After H1N1 influenza virus (PR8 strain) infection, inflammasome activation leads to the migration of DCs from the lungs to the draining lymph nodes and initiates T cell activation (179). Moreover, metabolic interactions between the gut and lungs may influence the progression of influenza virus infection by regulating pathways like glycolysis (180).

### 5.2 Regulatory role of the gut-lung axis in RSV infection

RSV is a single-stranded, negative-sense RNA virus and a significant pathogen of lower respiratory tract infections, especially in infants and immunocompromised individuals. Severe cases can lead to bronchiolitis or pneumonia. Studies have shown that the gut microbiota plays a critical role in modulating immune responses to RSV infection, and dysbiosis of the gut microbiome is closely related to the pathogenesis of RSV infection.

Studies have demonstrated that RSV infection not only significantly alters the diversity and abundance of the gut microbiota but also induces intestinal immune damage (169). In mouse models of RSV infection, research has found a decrease in beneficial bacteria and an increase in opportunistic pathogens (181–183). The populations of key bacterial groups in the gut microbiota, such as Lactobacillus and Bifidobacterium, undergo significant changes, which in turn affect pulmonary immune responses and inflammation levels. RSV infection increases the expression of specific immune-regulatory cells and cytokines in the gut, which may help control viral replication and the inflammatory response in the lungs. For example, RSV infection leads to an initial increase, followed by a decline, in the mRNA levels of ROR-γt and Foxp3 in both the lungs and gut (169). These genes are closely related to the differentiation and function of Tregs and Th17 cells (184, 185), which play crucial roles in maintaining immune balance and regulating lung inflammatory responses (169, 186). During infection, the expression of pro-inflammatory cytokines and chemokines, such as TNF-α, IL-6, and IFN-γ, increases in the gut, which may help inhibit viral replication and alleviate lung inflammation (187). Chemokine ligand 4 (CXCL4) inhibits RSV replication by binding to heparan sulfate, the primary RSV receptor, thereby blocking viral attachment (188). Additionally, RSV infection activates the host IFN-I signaling pathway, inducing the expression of ISGs such as MX1 and OAS1, which suppress viral replication (189). Moreover, metabolites play a significant role in immune regulation within the gut-lung axis. Acetate has been demonstrated to protect mice from RSV infection in an IFNAR-dependent manner. Through IFNAR mediation, the activation of GPCRs such as GPR43 in alveolar epithelial cells reduces virus-induced cytotoxicity and enhances antiviral effects via the IFN-β response (190). Thus, by modulating the gut microbiota or targeting specific immune pathways within the gut-lung axis, it may be possible to improve airway inflammation and lung damage caused by RSV infection, thereby influencing the prognosis (191–193).

### 5.3 Regulatory role of the gut-lung axis in SARS-CoV-2 infection

SARS-CoV-2, a positive-sense single-stranded RNA virus, is a novel respiratory pathogen that has caused the global COVID-19 pandemic, leading to a significant public health burden worldwide. Recent studies have revealed the critical role of the gut-lung axis in regulating immune responses during SARS-CoV-2 infection, primarily through its systemic immune modulation function.

Studies indicate that the gut microbiota can influence the expression of ACE-2, the primary receptor that mediates SARS-CoV-2 entry into host cells. ACE-2 plays a pivotal role in regulating inflammation and viral entry (194), and its expression is closely linked to the composition of the gut microbiota (195, 196). For instance, *Bacteroides* spp. can downregulate ACE-2 expression in the colonic epithelium, reducing viral binding efficiency (195). Dysbiosis of the gut microbiota has been observed in COVID-19 patients, potentially increasing susceptibility to the virus by upregulating ACE-2 expression (195–197).

The common reduction in gut microbiota diversity observed in COVID-19 patients also includes a decrease in anti-inflammatory bacterial genera, such as *Faecalibacterium prausnitzii*, and an enrichment of opportunistic pathogens, such as *Clostridium ramosum* and *Enterococcus (* 194, 195). This dysbiosis can exacerbate lung injury through various mechanisms, including a decrease in SCFAs production, which impairs M2 polarization of AMs and inhibits the secretion of anti-inflammatory cytokine IL-10 (195, 198); increased intestinal permeability, allowing PAMPs to enter the circulation and trigger a systemic “cytokine storm” (194); and dysregulation of hematopoietic function in the bone marrow, promoting an increase in Ly6C^+^ inflammatory monocytes and exacerbating lung tissue damage (195).

Moreover, further evidence from the newly developed humanized ACE-2 knock-in (hACE2-KI) mouse model suggests that changes in the gut microbiota during SARS-CoV-2 infection through the lung-gut axis may mitigate excessive inflammatory responses. An increase in certain bacterial genera, such as *Lachnospiraceae_NK4A136_group* and *unclassified_f_Lachnospiraceae*, may help alleviate the overactive inflammation induced by SARS-CoV-2 infection. Conversely, an increase in *Staphylococcus* species in fecal samples may indicate bacterial migration from the gut to the lungs, potentially triggering secondary infections in the lung-gut axis (199).

## 6 Potential applications of the gut-lung axis in antiviral therapy

**Figure 3:**
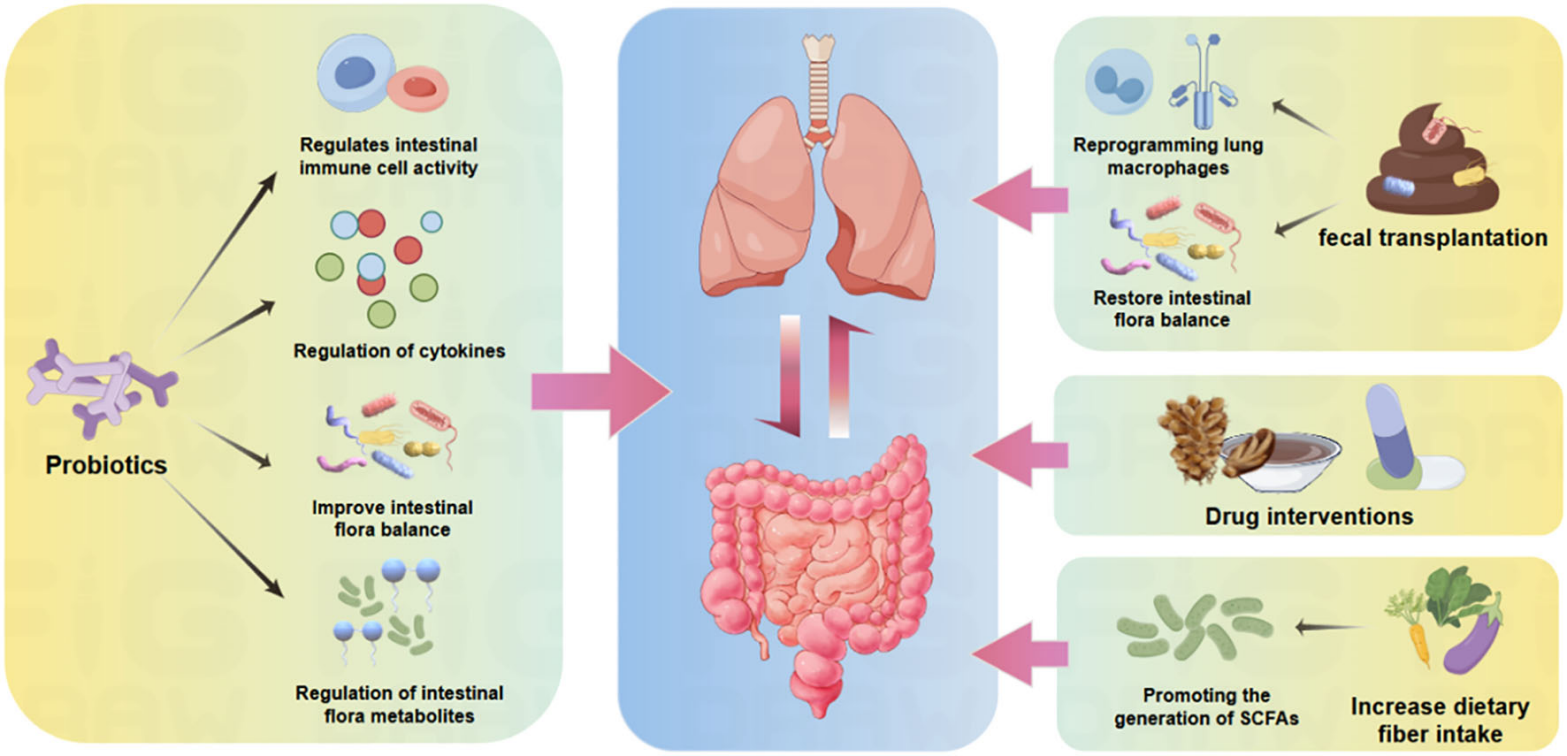
Anti-respiratory viral infection therapies targeting the gut-pulmonary axis. Probiotics can regulate intestinal immune cells, modulate cytokines, improve intestinal microbiota balance, and regulate intestinal microbiota metabolites; fecal transplants can reprogram lung macrophages on one hand, and restore intestinal microbiota balance on the other; pharmacological interventions (including TCM and western medicine) can regulate antiviral immune functions in the lungs and intestines; and increased intake of dietary fibers can promote the production of SCFAs, enhance intestinal antiviral immunity.

Numerous studies have shown that the gut-pulmonary axis can be a potential target for anti-respiratory viral infection therapy, and there are an increasing number of antiviral therapeutic regimens with the primary goal of modulating the immune function of the lungs and intestines, such as physician’s bacilli, dietary modification, and antiviral drug interventions. **Figure 3** summarizes several clinically used anti-respiratory viral infection therapeutic regimens targeting the gut-lung axis and their main roles, which are described in detail below.

### 6.1 The use of probiotics and prebiotics

Probiotics and prebiotics, as effective tools for modulating the gut microbiota, have been extensively studied. By increasing the number of beneficial bacteria or providing the nutrients required for their growth, they can regulate the intestinal immune environment, thereby influencing the antiviral immune response in the lungs.

*Bifidobacterium*, *Lactobacillus rhamnosus*, and *Lactobacillus plantarum* have shown significant effects in modulating excessive inflammatory responses. *In vitro* studies have demonstrated that probiotics can reduce the release of pro-inflammatory cytokines, prevent excessive activation of the NLRP3 inflammasome, mitigate inflammation, and reduce the risk of lung fibrosis caused by COVID-19 (200). *Bifidobacterium* can also enhance neutrophil phagocytic activity and (201), together with *Lactobacillus rhamnosus*, modulate T cell responses, improve gut microbiota balance, promote the production of antibodies such as IgA and IgG, and boost immunity against influenza viruses (202). *Lactobacillus* not only exerts its effects through antimicrobial mechanisms but also by modulating immune responses along the gut-lung axis, restoring the microbial balance between the gut and lungs, thereby alleviating symptoms such as ARDS induced by SARS-CoV-2 (200).

Studies have also found that the intake of probiotics significantly prevents and mitigates respiratory viral infections. For example, the consumption of appropriate amounts of *Lactobacillus plantarum* and *Lactobacillus paracasei* in adults has been shown to shorten the duration of the common cold, relieve symptoms, and enhance the immune response to influenza vaccination (203). Prebiotics, such as inulin and fructo-oligosaccharides, improve lung health indirectly by promoting the production of SCFAs, strengthening the gut barrier function, and reducing inflammatory responses.

### 6.2 Dietary regulation and microbiota transplantation

Dietary regulation, through the adjustment of fiber, fat, polyphenols, and other components in the diet, directly influences the composition and function of the gut microbiota, thereby enhancing antiviral immune responses. For example, increasing dietary fiber intake not only promotes the production of SCFAs but also strengthens antiviral immunity. In traditional Chinese medicine (TCM), the concept of “the gut and lungs sharing a common origin” suggests a close connection between intestinal health and respiratory diseases, implying that gut health has a potential link to respiratory conditions, This aligns with the modern medical concept of the “gut-lung axis” (204). Based on this foundation, certain Chinese medicines, such as *Qinbai Qingfei Concentrated Pill* and *Xuanfei Baidu Formula*, have been used to alleviate pulmonary inflammation and modulate immune responses, exhibiting bidirectional regulatory effects on the gut-lung axis. These treatments not only reduce lung inflammation but also restore systemic immune balance by modulating the gut microbiota (205).

Fecal microbiota transplantation (FMT), an emerging microbial transplantation technology, has also shown preliminary potential in treating respiratory viral infections through regulation of the gut-lung axis. FMT can restore gut microbiota balance, reduce lung inflammation, and enhance antiviral immunity. Research has demonstrated that FMT can reprogram lung macrophages, improving their efficacy in combating respiratory viruses (154).

### 6.3 Drug intervention and regulation of gut microbial metabolites

Certain antiviral drugs not only act directly on viruses but also indirectly modulate the host immune response by affecting the gut microbiota. Studies have shown that influenza virus infection can lead to gut microbiota imbalance, while rifaximin, a non-absorbable antibiotic, significantly increases the abundance of *Lactobacillus* and *Bifidobacterium*, restoring gut microbial balance. It also reduces tissue damage by strengthening lung and intestinal barrier function (206).

Moreover, promoting the production of SCFAs or related metabolites, such as indole-3-propionic acid (IPA), has shown great potential in antiviral therapy. Research indicates that specific probiotic mixtures can reverse gut dysbiosis caused by RSV infection and significantly increase SCFA levels. The elevation of SCFAs enhances the antiviral capabilities of immune cells in the lungs via the gut-lung axis, thereby boosting antiviral immune responses (191). In another study, mice infected with IAV treated with probiotics exhibited a marked increase in butyrate levels, a reduction in viral load, and an enhanced immune response (207). This suggests that promoting SCFA production could be a key therapeutic strategy in mitigating viral infections through the gut-lung axis. Additionally, supplementation with metabolites such as IPA has been shown to reduce influenza viral load and alleviate both pulmonary and systemic inflammation. Therefore, therapies aimed at boosting the production of SCFAs and IPA may represent promising approaches for preventing and treating respiratory viral infections in the future (208).

## 7 Conclusion and perspectives

Despite increasing research highlighting the critical role of the gut-lung axis in various respiratory viral infections, there remain several limitations. First, the precise molecular mechanisms and their interactions are not yet fully understood, particularly concerning the role of SCFAs in pulmonary immune regulation, where variability persists (209). Second, in clinical diagnosis and treatment, it remains challenging to accurately determine whether a disease is linked to dysregulation of the gut-lung axis, and the effectiveness and applicability of related interventions require further clinical validation. Additionally, constructing experimental models that accurately reflect human conditions poses technical challenges, and existing models may diverge from real-world scenarios, potentially affecting the accuracy of research findings (210, 211).

Looking ahead, future research should focus on elucidating how the gut microbiota modulates pulmonary immune responses through specific molecular pathways. The integration of multi-omics technologies, such as single-cell sequencing, metabolomics, and proteomics, will help uncover the key molecules and signaling pathways within the gut-lung axis. Identifying functional microbial strains related to pulmonary immune regulation, along with in-depth studies using gene-editing technologies, will advance personalized therapeutic strategies. As research progresses, personalized antiviral therapies based on the gut-lung axis are likely to achieve breakthroughs. Moreover, the roles of other inter-organ axes, such as the gut-brain, gut-liver, and lung-brain axes, in respiratory viral infections also warrant further exploration. By integrating these complex networks, future research will provide a more comprehensive theoretical foundation for the prevention and treatment of viral infections.
